# Characterization of Progesterone Receptor Expression in Intracranial Meningiomas of Patients Treated in a High-Complexity Hospital in Bogota, Colombia

**DOI:** 10.7759/cureus.12355

**Published:** 2020-12-29

**Authors:** Raul Ramirez Grueso, Linda Barcenas, Jaime A Arias, Carlos Colegial, Claudia L Avendaño, Jose Chaves, Jorge Galvis, Santiago Moreno

**Affiliations:** 1 Department of Neurosurgery, National University of Colombia, Bogota, COL; 2 Department of Pathology, National University of Colombia, Bogota, COL; 3 Inmunohistochemistry/Electron Microscopy, Bio-Molecular Diagnostica, Bogota, COL; 4 Department of Neurosurgery, Santander University Hospital, Bogota, COL; 5 Department of Neurosurgery, Subred Integrada de Servicios de Salud Sur Occidente, Bogota, COL

**Keywords:** meningioma, progesterone receptor, ki67

## Abstract

Background

Meningiomas are the single most common brain tumor. The incidence of these tumors increases with age; different studies have shown that meningiomas usually appear after the age of 50. These tumors are more common in women than in men, and women are twice as likely to suffer from the condition. Surgery is the primary form of treatment, which can be curative with complete resection. If the tumor is unresectable or other treatments such as surgery and radiotherapy have failed, hormonal therapy or chemotherapy may be considered. There is limited information about the clinical, demographic, and histopathological characteristics of these tumors in the population of Bogotá, Colombia.

Objective

To evaluate the expression of progesterone receptors in patients over 18 years old who have been diagnosed with meningiomas in a high-complexity hospital in Bogota, Colombia, and to describe the demographic and histopathological characteristics of these patients.

Methods

This is a descriptive and retrospective case series. Patients with meningioma who underwent surgical resection at a high-complexity hospital in Bogota, Colombia, from 2016 to 2019 were retrospectively identified and studied. Demographic variables, such as age and gender, were extracted from the clinical chart. Indirect immunoperoxidase staining was carried out for the progesterone receptor (PR) and Ki67. PR is analyzed as positive and negative, and the Ki67 proliferation index was determined.

Results

Thirty-two meningiomas from patients who underwent surgery were available for analysis. Twenty-five (78.1%) were positive for PR, 71.8% were females, and 93% were World Health Organization (WHO) grade I. Meningothelial (28%), fibrous (25%), and transitional (25%) meningiomas were the most frequent subtypes, correspondingly. The Ki67 mean value was 1.14 (0.11-10.71).

Conclusion

Our case series showed a greater frequency of meningiomas in women, with a high PR expression and a low Ki67 proliferation rate. These data correlate with literature worldwide.

## Introduction

Meningiomas are the single most common brain tumor, comprising approximately 36.5% of all primary brain tumors [1-2]. These tumors form and grow from the arachnoid cap cells found at the arachnoid villi and can be found anywhere in the skull vault, skull base, or spinal canal [3]. Different studies have shown that meningiomas appear more frequently in later life, particularly after the age of 50. Furthermore, these tumors are more common in women who are twice as likely as men to suffer from the condition if both genders are compared [4-5]. The annual incidence worldwide per 100,000 people ranges from 2 to 7 for females and 1 to 5 for males [3-5]. Ionizing radiation to the skull is considered a risk factor for the development of meningioma, with a six to tenfold relative risk following a variable latency period and without a clear dose-response relationship [4-5]. History of head trauma, cigarette smoking, and mobile phone use has not been consistently shown to be associated with a significantly increased risk of meningioma. Several familial syndromes predispose to meningioma development, with the most common hereditary cause being neurofibromatosis type 2 (NF2), also including Li-Fraumeni, Gorlin, von Hippel-Lindau, Cowden disease, and multiple endocrine neoplasia (MEN) type 1 [6-9]. The 2016 World Health Organization (WHO) classification of central nervous system tumors distinguishes 15 histological variants of meningioma [10-11]. Molecular profiling efforts of sporadic benign meningiomas have identified two, large, molecularly and clinically divergent clusters: a clinically heterogeneous group of tumors with mutations of the NF2 gene and/or loss of chromosome 22; and non-NF2 meningiomas with frequent alterations in the Sonic hedgehog (Shh) pathway, phosphatidylinositol-3-kinase (PI3K) signaling pathway, and TRAF7, KLF4, or POLR2A genes, which are clinically benign and often occur at the base of the skull [11-13].

Like other central nervous system (CNS) tumors, the presentation of meningiomas depends upon their location. Meningiomas are typically not fast-growing or infiltrative lesions, and they have an insidious symptom onset. Many are discovered incidentally on brain imaging [10,14]. Several observational studies have shown a linear growth rate of 2-4mm/year for asymptomatic meningioma [12].

There are different options to treat meningioma; out of these, surgery is the most common option when it can be fully removed since it can represent complete recovery for the patient. Treatment options for meningioma have been widely described in the literature; chosen treatments depend on the location of the tumor on the brain [3]. In addition to surgery, other treatments include radiotherapy, hormonal therapy, and chemotherapy, any of which can be applied to the patient depending on their particular conditions at the clinical assessment time [3]. To identify the safest and most effective treatment options for each patient, an analysis of possible pre-, intra-, and postoperative complications that can arise is performed [1,3,12].

To date, there is limited information about the demographic, clinical, and histopathological characteristics of patients with these tumors in our population. Therefore, our objective is to evaluate the expression of progesterone receptors in patients diagnosed with meningiomas in a high-complexity hospital in Bogota, Colombia, and to describe the demographic and histopathological characteristics of this population.

## Materials and methods

We conducted a descriptive and retrospective case series on patients with intracranial meningioma who underwent surgical treatment at a high-complexity hospital between January 1, 2016, and June 30, 2019. This search yielded 51 patients, from which only 32 had enough tissue to take for immunohistochemical analysis and possessed comprehensive clinical data, which were selected and studied. Inclusion criteria were: 18 years old or over, a histopathological diagnosis of intracranial meningioma, and surgical treatment for tumor biopsy or resection. Exclusion criteria were insufficient tissue for the immunohistochemical analysis (n=7) and incomplete clinical data (n=12).

Data related to clinical history, neuroimaging, and surgical procedure were extracted from the clinical chart. The variables analyzed included age, gender, location of the tumor on imaging, grade of tumor resection according to Simpson's grading system, histopathological subtype, and grade of tumor according to the 2016 CNS WHO classification. Indirect immunoperoxidase staining was carried out for PR and Ki67. PR was analyzed as positive and negative, and the Ki67 proliferation index was determined through the Aperio imaging score software (Leica Biosystems, Wetzlar, Germany). A total of 7360 nuclei were analyzed on average for each case, and only nuclei with +3 staining were counted. Descriptive statistical analysis was carried out using the Statistical Package for the Social Sciences (SPSS) version 26 software (IBM Corp., Armonk, NY).

## Results

Thirty-two (32) meningiomas from patients that underwent surgery were available for analysis; 71.8% were females; 25 patients were positive for PR and split by gender; and PR was positive in 78.3% of females and 77.8% of men. The age distribution is presented in Table 1.

**Table 1:** Age distribution

Age	No. of cases
20 - 29	2
30 - 39	2
40 - 49	8
50 - 59	8
60 - 69	10
70 - 79	1
80 - 89	1
Total	32

**Figure 1:**
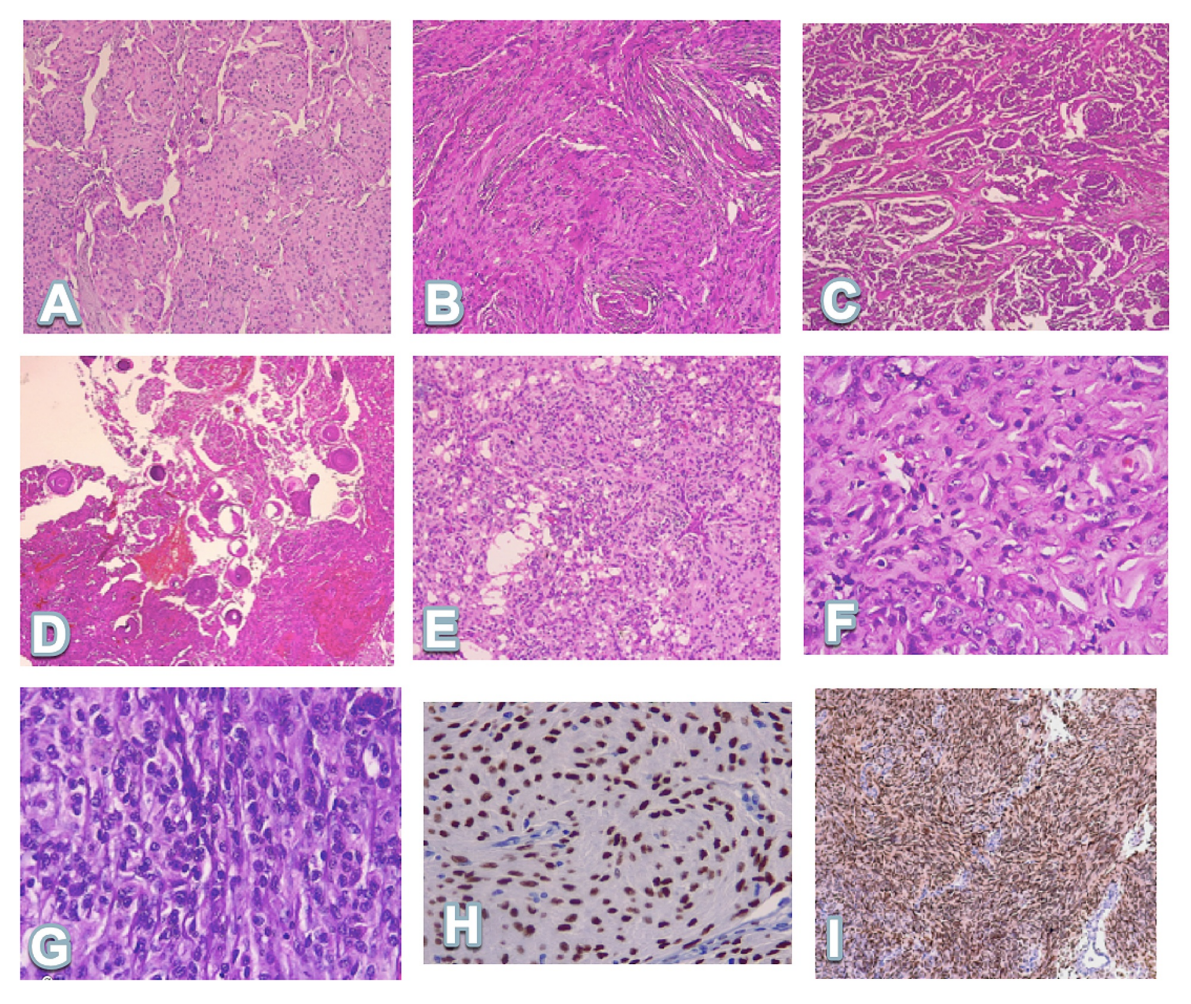
The most frequent histological variants A) Meningothelial, B) Fibrous, C) Transitional, D) Psammomatous, E) Microcystic, F) Atypical, G) Anaplastic, H) Image of immunohistochemical staining with the progesterone receptor antibody in a case of meningioma meningothelial, I) Image of immunohistochemical staining with the progesterone receptor antibody in a case of meningioma fibrous

Regarding tumoral grade, 93% were WHO grade I, 3.1% WHO grade II, and 3.1% WHO grade III, correspondingly. The distribution regarding histopathological variants was: meningothelial (28%), fibrous (25%), transitional (25%), psammomatous (3.1%), microcystic (3.1%), atypical (3.1%), anaplastic (3.1%), and undefined (9.4%). The most recurrent tumor location was parasagittal (43.8%), followed by the sphenoid ridge (18.8%), infratentorial (18.8%), olfactory groove (9.4%), brain convexity (6.3%), and intraventricular (3.1%), respectively (Figure 1). Tumors were located according to the case as follows: in 34.4% of the cases, on the right side; in 37.5%, on the left side, and in 28.1%, on the midline. The distribution of meningiomas according to location is presented in Table 2.

**Table 2:** Distribution of meningiomas according to location

Locations		No. of cases
Medial skull base	Olfactory groove	3
	Total	3
Lateral skull base	Sphenoid ridge	6
	Total	6
Non-skull base	Cerebral convexity	2
	Parasagittal	14
	Intraventricular	1
	Infratentorial	6
	Total	23
Total		32

There were three cases of medial skull base meningiomas, and two of them were positive for PR. We found six lateral skull base meningiomas, out of which 83.3% were positive for PR, and 23 non-skull base meningiomas, from which 78.3% were positive for PR. Eighty-one point twenty-five percent (81.25%; n=26) meningiomas occurred in the supratentorial compartment and 18.75% (n=6) occurred in the infratentorial compartment. The study evidences the following distribution for Simpson grade resections: grade I (59.3%), grade II (6.3%), grade III (9.4%), and grade IV (25%). The Ki67 mean value was 1.14 (0.11-10.71) and the cases with the highest Ki67 proliferation index are presented in Table 3.

**Table 3:** Illustrates the main clinicopathological characteristics of patients with a proliferation index greater than 3% PR: progesterone receptor; WHO: World Health Organization

Case	Gender	Age	Location	Histological variant	WHO grade	PR	Ki67
1	M	57	Infratentorial	Psammomatous	I	Positive	4.01%
2	M	68	Parasagittal	Meningothelial	I	Positive	4.21%
3	H	37	Sphenoid ridge	Transitional	I	Positive	4.01%
4	H	61	Parasagittal	Fibrous	I	Positive	5.73%
5	H	65	Convexity	Meningothelial	I	Negative	4.35%
6	M	53	Sphenoid ridge	Psammomatous	I	Positive	10.71%
7	M	43	Infratentorial	Meningothelial	I	Positive	4.60%

## Discussion

Since the early 1980s, the association of sex hormone receptors with meningiomas has been the subject of numerous studies [1,15-16]. The higher incidence of these neoplasms in women than in men, the relapse and remission of symptoms during pregnancy, and the epidemiological link described between meningiomas and breast carcinomas have led to the assumption that the hormonal environment might influence the growth of these tumors [5-6,13,17-19].

Several studies have focused on the presence or absence of the PR and the estrogen receptor (ER) in meningiomas; therefore, it has been documented that these types of tumors often contain steroid hormone receptors [17,19-20]. Nonetheless, the correlation between the receptor presence and the mitotic index with the patient prognosis continues to be studied [1,6-7,15].

Even though there is a general agreement that most meningiomas contain PR and are devoid of ER, conclusions about the importance of the receptor expression status in meningioma progression are still limited [20]. On the other hand, attempts to administer hormonal therapy for meningioma patients and the in vitro hormone manipulation in meningioma cells cultures have produced non-conclusive results [1,3,7,17].

Research is being done to evaluate the potential of manipulating the tumor's hormonal environment as a valid intervention method to provide multiple therapeutic options for patients with pathologies such as meningioma [21-23].

Findings in this study reveal similar results as compared to case series published in other countries. This study indicated that meningiomas were most common in the seventh decade, with 31.25% of occurrences, followed by the fifth and sixth decades, respectively, with 25% of cases in each one, which was a trend observed by Marossi and coworkers [2,15]. We found a higher frequency in females, with a female-male ratio of 3:1 to 2:1, slightly higher than other studies [2,4-5]. We also found that meningiomas are more frequent in the supratentorial compartment, with about 90% of occurrences, especially in the lateral skull, which has been a constant finding in several studies [4-5,7,13,23-26]. In this study, 25 of 32 meningiomas (78%) were PR positive and more frequent in women (72%, n=18/25). In the past 15 years, several studies have published similar results [2,15]. One study found that medial skull base meningiomas had the highest PR expression as compared to lateral skull base and non-skull base meningiomas [26]. Our case series found that lateral skull base meningiomas was the group with the highest prevalence of PR receptors, followed by non-skull base, and finally medial skull base meningiomas.

According to the histological subtype, we found that meningothelial (28%), fibrous (25%), and transitional (25%) were the most frequent subtypes, finding similar results when compared with other studies [26].

Medial skull base meningiomas were found to be from the meningothelial type in 100%. The histopathological types found were 50% transitional for the lateral skull base group and 16.6% each corresponded to meningothelial, fibrous, and psammomatous.

Non-skull base meningiomas were mainly from the fibrous type, n=7 (30.4%), and n=5 (21.7%) corresponded to meningothelial and transitional, one each. Compared to the study carried out by Maiuri and coworkers [26], they found that the most prevalent type of meningioma in medial, lateral, and non-skull base was transitional.

Our study indicated that 93% (30 of 32) of meningiomas are WHO grade I, 3.3% (one of 32) are WHO grade II and 3.3% (one of 32) are WHO grade III. When compared to other studies, ours revealed a higher prevalence of WHO grade I meningiomas, lower grade II, and similar grade III [2-3,15,27]. Furthermore, non-skull base tumors had higher rates of WHO grade II and III tumors than medial and lateral-skull base tumors. On the other hand, Maiuri and coworkers found that lateral skull base was the group with higher WHO II and III rates [26]. Table 4 shows the correlation between PR status and WHO grade.

**Table 4:** Correlation between PR status and WHO grade PR: progesterone receptor; WHO: World Health Organization

PROGESTERONE RECEPTOR	WHO CLASSIFICATION	
POSITIVE	GRADE I	24 (96%)
	GRADE III	1 (4%)
	TOTAL	25
NEGATIVE	GRADE I	6 (85.7%)
	GRADE II	1 (14.3%)
	TOTAL	7

In other studies, it was found that atypical and anaplastic meningiomas might be more common in males, possibly due to the higher proliferation indexes discovered in male-patient meningiomas [2,16]. In our series, there was one atypical case found in a woman and one anaplastic meningioma found in a male.

We found n=12 (37.5%) left-located meningiomas, 11 (34.3%) right-located meningioma,s and nine located in the midline (28.2%). All right tumors were PR positive. We did not find literature comparing the laterality of the tumor and its association with being positive for progesterone. More research is needed on this topic. In our series, the Ki67 mean value was 1.14, similar to the results reported in Maiuri and coworkers' series [26]. Table 5 shows the correlation between tumor location and ki67.

**Table 5:** Correlation between tumor location and ki67

Tumor location		Ki67		
	Number of cases	Minimum	Maximum	Mean
Convexity	2	0.97	4.02	2.4937
Parasagittal	13	0.23	5.73	1.7824
Sphenoid ridge	6	0.57	10.71	3.7731
Intraventricular	1	0.23	0.23	0.2287
Infratentorial	6	0.21	1.45	0.9892
Olfactory groove	3	0.11	1.67	0.8626

The study evidences the following distribution for the Simpson grade of resections: grade I (59.3%), grade II (6.3%), grade III (9.4%), and grade IV (25%). Series published in other countries found that for patients with a Simpson grade I resection, the meningioma has a 10-year recurrence rate of 9% as compared with patients with a Simpson grade 3 resection, for whom meningioma recurrence rate is 29% over the same 10-year period [27]. Jaaskelainen and coworkers found a recurrence rate of 19% at 20 years, following complete resection of WHO grade 1 tumors. They reported that for patients with atypical or malignant meningiomas following complete resection, the recurrence risk was 38% and 78%, respectively, after five years [16]. Our study did not evaluate recurrence because of the slow growth rate of meningioma; less than 10 mm per year. Our series was a four-year study, and Simpson grade I resection was achieved in most cases. Likewise, we could have seen the recurrence of the WHO grade II and III meningiomas if our cohort had more years of follow-up.

## Conclusions

For the most part, meningioma patients treated with modern surgical and radiation therapies achieve successful outcomes. Unfortunately, many of these tumors are not amenable to complete surgical resection, and the possibility to control the tumor’s hormonal environment is still an alternative therapeutic target in the process of development. Numerous pharmacological treatments that focus on blocking progesterone's effect on meningiomas PR are being researched and developed worldwide. Therefore, it is essential to understand how the local population compares to other populations. This case series studied a heterogeneous and diverse sample of patients with meningiomas in a high-complexity hospital with one of the highest volumes of patients with this pathology in the country. It furthers our understanding of meningiomas' behavior in the local population, allowing us to evaluate if international research on pharmacological treatments could be extrapolated locally. Treatment through pharmacological PR inhibitors could allow patients to avoid repetitive surgeries. Our case series suggested greater frequency in women, high progesterone receptor expression, preponderant localization on the lateral skull base, and a low Ki67 proliferation rate. This data correlates with literature worldwide. Future studies with a large cohort and a more extended follow-up period are required for a thorough demographic characterization of meningiomas in this region.
